# Supplementary material for: Porphyromonas gingivalis Lipopolysaccharides Promote Proliferation and Migration of Human Vascular Smooth Muscle Cells through the MAPK/TLR4 Pathway
Source: Int J Mol Sci. 2022 Dec 21;24(1):125. doi: 10.3390/ijms24010125 (PMC9820797; doi:10.3390/ijms24010125)
Supplement: Supplementary file 1 [file ijms-24-00125-s001.zip › supplemental figure.pdf]

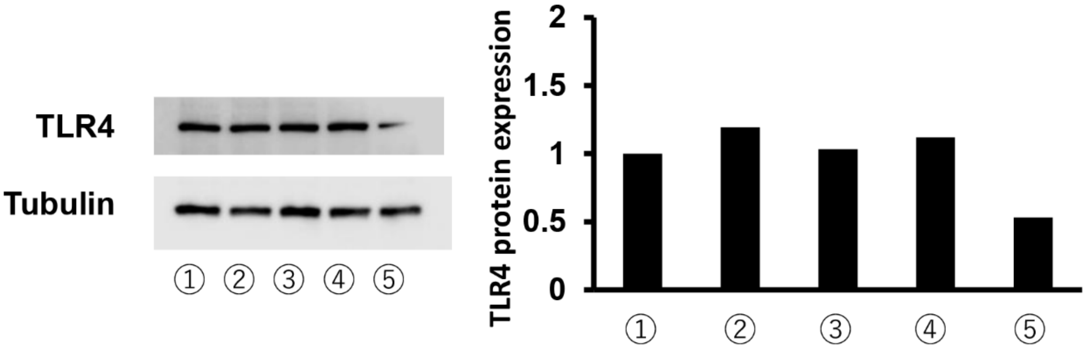

Supplemental Figure S1. Knockdown efficacy of siRNAs of TLR4. For short interfering (si) RNA transfection, HASMCs, after reaching 70-80% confluence, were transfected with siRNAs of TLR4 (Qiagen, Venlo, Netherlands) using HiPerFect (QIAGEN, Venlo, Netherlands).

①:non transfection control, ②:mock transfection control(HiPerFect + DMEM), ③:non-silencing RNA, ④,⑤: siRNAs of TLR4, SI04951149, SI00151011, respectively. We decided to knock down TLR4 using TLR4 siRNA (SI00151011). TLR4 protein expression is suppressed by 49 %.

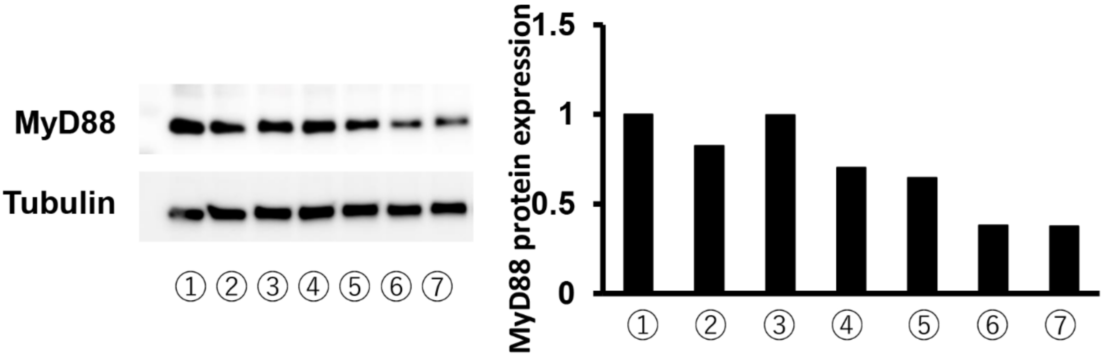

Supplemental Figure S2. Knockdown efficacy of siRNAs of MyD88. For short interfering (si) RNA transfection, HASMCs, after reaching 70-80% confluence, were transfected with siRNAs of MyD88 (Qiagen, Venlo, Netherlands) using HiPerFect (QIAGEN, Venlo, Netherlands).

①:non transfection control, ②:mock transfection control(HiPerFect + DMEM), ③:non-silencing RNA, ④,⑤,⑥,⑦: siRNAs of MyD88, SI00300909, SI05426057, SI03100902, SI02628661 respectively. We decided to knock down MyD88 using MyD88 siRNA (SI02628661). MyD88 protein expression is suppressed by 61%.
